# Induction of Ferroptosis by Ophiopogonin-B Through Regulating the Gene Signature *AURKA* in NSCLC

**DOI:** 10.3389/fonc.2022.833814

**Published:** 2022-06-28

**Authors:** Liqiu Li, Qian Gao, Jin Wang, Ling Gu, Zhihui Li, Shiping Zhang, Cheng Hu, Menglin He, Yulin Wang, Zixuan Wang, Yongxiang Yi, Jin Fu, Xiongfei Zhang, Fei Ge, Meijuan Chen, Xu Zhang

**Affiliations:** ^1^ School of Medicine & Holistic Integrative Medicine, Nanjing University of Chinese Medicine, Nanjing, China; ^2^ The Second Hospital of Nanjing, Nanjing University of Chinese Medicine, Nanjing, China; ^3^ College of Traditional Chinese Medicine & Integrated Chinese and Western Medicine College, Nanjing University of Chinese Medicine, Nanjing, China; ^4^ The First Clinical Medical College, Nanjing University of Chinese Medicine, Nanjing, China; ^5^ Department of Gastroenterology, Haian Hospital of Traditional Chinese Medicine, Nantong, China

**Keywords:** ophiopogonin-B (OP-B), ferroptosis, *AURKA*, NSCLC, bioinformatics

## Abstract

Ferroptosis is a new type of iron-dependent programmed cell death. In recent years, its role in the diagnosis and treatment of multiple tumors, including non-small cell lung cancer (NSCLC), has been continuously observed. The relationship between the ferroptosis-related genes and the prognosis of patients with NSCLC needs to be clarified. In this study, The Cancer Genome Atlas (TCGA) and the Gene Expression Synthesis database (Gene Expression Omnibus, GEO) were used to build a model of ferroptosis-related differentially expressed genes (DEGs). A total of 101 ferroptosis-related DEGs were screened using R language, and a 12-gene signature was finally established through univariate Cox regression analysis and least absolute shrinkage and selection operator (LASSO)-penalized Cox regression analysis. According to the risk scores, the patients were divided into a high-risk or a low-risk group, with patients in the low-risk group showing better prognosis. *AURKA*, one of the genes in the 12-gene signature, was found to be highly expressed in tumors. In addition, further study verified *AURKA* to be a negative regulator of ferroptosis in NSCLC cells. Ophiopogonin B (OP-B) had been reported to induce apoptosis, mitotic catastrophe, and autophagy in NSCLC cells. Herein, proteomic sequencing analysis and OP-B administration revealed the upregulation of *AURKA* and the downregulation of *PHKG2* and *SLC7A5* in the 12-gene signature, indicating that OP-B induced ferroptosis in NSCLC. Determination of the concentrations of malondialdehyde (MDA), glutathione (GSH), and intracellular iron and the mitochondrial membrane potential (MMP) confirmed the induction of ferroptosis by OP-B *in vitro*. Furthermore, transmission electron microscopy (TEM) examination of lung cancer xenotransplantation in nude mice confirmed that OP-B induced ferroptosis *in vivo*. Further study of the molecular mechanism showed that the ferroptosis effect caused by OP-B can be partially reversed by the overexpression of *AURKA*. Overall, our study established a new ferroptosis-related risk prediction model for the prognosis of patients with NSCLC, revealed the enrichment pathways of ferroptosis in NSCLC, and discovered the negative regulation of *AURKA* in ferroptosis. On this basis, we demonstrated that OP-B can induce ferroptosis in NSCLC and clarified the specific molecular mechanism of OP-B inducing ferroptosis by regulating the expression of *AURKA*.

## Introduction

Non-small cell lung cancer (NSCLC) accounts for almost 80% of all lung cancers, including lung adenocarcinoma (LUAD), lung squamous cell carcinoma (LUSC), and large-cell carcinoma. Patients with NSCLC are often found to have distant metastases at an early stage, even after chemotherapy, and the 5-year survival rate is only 5% (1). Because of the anti-apoptotic characteristics of tumor cells, researchers started to explore new antitumor strategies. The occurrence and the development of tumors are often accompanied by an imbalance of the redox environment and a high demand for iron ions, suggesting that tumor cells are highly sensitive to ferroptosis (2). Therefore, discovering drugs that induce the ferroptosis of tumor cells has become a hot research direction.

Ferroptosis is a form of cell death mainly characterized by a series of mitochondrial morphological changes, including mitochondrial shrinkage, reduced or disappeared mitochondrial cristae, increased mitochondrial membrane density, and ruptured outer membranes of the mitochondria, among others (3). The initiation of ferroptosis is a sophisticated process involving increased intracellular divalent iron, accumulation of lipid peroxide (LOOH), and imbalance in the generation and clearance of reactive oxygen species (ROS) (4). At present, studies on the mechanism of ferroptosis mostly focus on the following pathways: 1) exhaustion of glutathione (GSH) and reduction in the activity of glutathione peroxidase 4 (GPX4), the GSH/GPX4 pathway (5), and 2) reduction in the activity of ferroptosis suppressor protein l (FSP1) and exhaustion of CoQ10, the FSP1/CoQ/NADPH pathway (6, 7). In addition, ferroptosis is regulated by multiple factors, such as Fe ions, glutamate/cystine transporter (system Xc-), P53, hypoxia inducible factor-1 α (HIF-1α), nuclear factor erythroid 2-related factor 2 (Nrf2), and heat shock proteins (HSFs), among others (5, 8–12). All of these regulatory factors could affect the susceptibility of cancer cells to ferroptosis. As the levels of ROS in cancerous cells are always higher than those in normal cells, ferroptosis has become a new treatment strategy for malignant tumors.

Currently, several clinical drugs, such as sorafenib, artemisinin, and sulfasalazine (SASP), have been confirmed to induce ferroptosis in tumor cells (13, 14). In addition, experimental research drugs inducing ferroptosis have also been emerging in recent years, such as erastin, acetaminophen (APAP), and ferroptosis-inducing agents (FINs) (15–17). However, a lot of studies have reported that the inhibition of ferroptosis is ubiquitous in lung cancer cells; for example, xCT (*SLC7A11*), serine/threonine/tyrosine kinase 1 (*STYK1*), and iron–sulfur cluster biosynthetic enzyme (*NFS-1*) have been found to be highly expressed in NSCLC cells (18–20). Therefore, exploration of the repressive or contributory ferroptosis-associated molecules in NSCLC cells is of great significance to develop the therapeutic effect of ferroptosis in NSCLC.

In this study, based on The Cancer Genome Atlas (TCGA) and the Gene Expression Omnibus (GEO), we extracted the ribonucleic acid (RNA) sequence datasets and clinical information of LUSC and LUAD and used differentially expressed genes (DEGs) related to ferroptosis to construct a 12-gene signature in order to predict the prognosis of patients with NSCLC. Moreover, pathway enrichment analysis was performed to explore the molecular mechanism of potential ferroptosis in NSCLC. It is worth noting that Aurora kinase A (*AURKA*), a cell cycle-related oncogene, was also included in the ferroptosis-related gene signature model. While previous research on *AURKA* mainly focused on cell proliferation, its role in ferroptosis needs to be verified.

Ophiopogonin B (OP-B), a saponin compound isolated from *Ophiopogon japonicus* (Thunb.), has multi-target antitumor characteristics (21–23). In this study, analyses of the protein Gene Ontology (GO) terms and the Kyoto Encyclopedia of Genes and Genomes (KEGG) pathways both exhibited OP-B as affecting the binding of intracellular ferric iron and regulating the gene expression in the ferroptosis-related pathway. In particular, OP-B was found to downregulate the expression of *AURKA* in the screening of the differentially expressed proteins.

Along with the increasing attention on ferroptosis in the diagnosis and treatment of NSCLC, some studies using the TCGA and GEO databases to establish NSCLC prognostic models have been reported in recent years (24–26). It is worth noting that we conducted a series of cytological experiments on the vital gene in the prognostic model based on prospective research and further identified its specific role in ferroptosis in NSCLC. Moreover, through proteomic sequencing, we combined the antitumor mechanism of the natural product OP-B with bioinformatics prediction, thus forming a relatively comprehensive research strategy (from bioinformatics prediction to verification with *in vivo* and *in vitro* experiments). In this study, we aimed to identify the role of *AURKA* in ferroptosis and to investigate the molecular mechanism of OP-B in inducing ferroptosis by regulating the expression of *AURKA* in NSCLC.

## Materials and Methods

### Data Collection

Using FerrDb (http://www.zhounan.org/ferrdb), 214 human orthologous genes were selected out of 259 genes (27). FerrDb is a manually curated database of ferroptosis-related markers, regulatory factors, and diseases, and the annotation datasets in FerrDb fall into three categories: drivers, suppressors, and markers. Ferroptosis affects the progression of diseases in two ways: either aggravating or alleviating. The raw RNA sequencing (RNA-seq) data and the clinical characteristics of 1,027 patients with LUAD and LUSC were obtained from TCGA (https://portal.gdc.cancer.gov/), which included 1,037 tumor tissue samples and 108 normal tissue samples. The microarray dataset GSE37745 was obtained from the GEO (https://www.ncbi.nlm.nih.gov/geo/) for validation, which contained 196 samples and patients’ clinical information.

To independently analyze the prognostic value of *AURKA*, raw counts of RNA-seq data (level 3) and corresponding clinical information (**Supplementary Table S1**) were downloaded from the TCGA dataset (https://portal.gdc.cancer.gov/), which included 252 NSCLC and 252 paracancerous samples. The messenger RNA (mRNA) expression of *AURKA* in normal and matched normal cancer samples of 24 tumors was analyzed on UALCAN (University of Alabama at Birmingham Cancer Data Analysis portal; http://ualcan.path.uab.edu/). The Human Protein Atlas (HPA; https://www.proteinatlas.org/about/download) was used to verify the mRNA sequencing data of *AURKA* across different cell lines and immunohistochemistry staining of genes. Data from the LUAD cell lines were downloaded from the Cancer Cell Line Encyclopedia (CCLE; https://portals.broadinstitute.org/ccle/).

### Screening of Differentially Expressed Ferroptosis-Related Genes

All ferroptosis-related genes were extracted from the ferrDb database, and we combined this gene set with the TCGA and GEO datasets to filter the ferroptosis-related gene expression of samples from the two databases. Calibration of the data from the two datasets was implemented through the “sva” package in *R* (4.0.3). To identify the DEGs related to ferroptosis, we performed differential expression analysis through the “limma” package in *R* (4.0.3). A *p*-value <0.05 was defined for DEGs.

### Construction and Validation of the Prognostic Gene Signature

Univariate Cox regression analysis was performed to screen and identify ferroptosis-related genes associated with overall survival (OS) in the training cohort. To minimize the risk of overfitting, least absolute shrinkage and selection operator (LASSO)-penalized Cox regression analysis was carried out to establish an NSCLC ferroptosis-related prognostic model using the “glmnet” package in *R* (28, 29). The patients’ risk scores were calculated according to the normalized expression level of each gene and its corresponding regression coefficients. We used the following formula to calculate the risk score for each patient:


Score = (normalized gene expression level × corresponding regression coefficient)

The median risk score was identified as the optimum cutoff value for classifying patients into the high-risk and low-risk groups.

### Kyoto Encyclopedia of Genes and Genomes Pathways on Gene Set Enrichment Analysis

Firstly, we imported the file containing the KEGG gene symbols into GSEA 4.1.0 and used the gene set enrichment analysis (GSEA) platform to perform functional enrichment analysis. The functional annotation of DEGs [|log2FC| ≥ 1, false discovery rate (FDR) < 0.05] in the prognostic risk models was described using the “cluster Profiler” *R* software package, which was utilized to conduct KEGG analyses (30).

### Samples and Reagents

OP-B (SZ20180901MDZGB) (HPLC ≥ 98%) was purchased from Nanjing Shizhou Bio-Technology Co., Ltd. (Shanghai, China). OP-B was dissolved in dimethyl sulfoxide (DMSO) as a 10-mM stock solution and stored at 4°C. For cell treatment, OP-B was diluted in a culture medium to the appropriate concentrations, and the final concentration of DMSO was <0.01%.

Matched human lung cancer and normal tissue samples were obtained during surgery. The study was conducted under informed consent and was approved by the Ethics Committee on Human Research of Haian Hospital of Traditional Chinese Medicine, Nantong, China. The study protocol followed the ethical guidelines of the 1975 Declaration of Helsinki (as revised in Brazil in 2013).

### Cell Culture

The A549 cell line was obtained from the Stem Cell Bank, Chinese Academy of Sciences (Shanghai, China). A549 cells were cultured in Dulbecco’s modified Eagle’s medium (DMEM)/F12 medium (Gibco, Melbourne, Australia) supplemented with 10% fetal bovine serum (FBS; Gibco, Melbourne, Australia), 100 U/ml penicillin, and 100 mg/ml streptomycin in incubators at 37°C with 5% CO_2_.

### Cell Transfection

According to the experimental arrangements, the A549 cells were assigned into the following groups: sh-NC group [treated with short hairpin RNA (shRNA) plasmid—negative control], sh-AURKA group (treated with the shRNA plasmids sh-AURKA-1 and sh-AURKA-2), p-NC group (treated with an *AURKA* overexpression plasmid—negative control), p-NC+OP-B group (treated with p-NC + 5 μM OP-B), p-AURKA group (treated with an *AURKA* overexpression plasmid), and p-AURKA+OP-B group (treated with p-AURKA + 5 μM OP-B). Cell transfection was performed with Lipofectamine 6000 transfection reagent (Beyotime, Shanghai, China) following the manufacturer’s protocol.

### Western Blot Analysis

The cells were lysed in radioimmunoprecipitation assay (RIPA) buffer (Beyotime, Shanghai, China) containing 1% phenylmethanesulfonyl fluoride (PMSF), and the protein concentration was detected with a BCA Protein Assay Kit (Beyotime, Shanghai, China). The proteins were separated on 10% SDS-PAGE (Beyotime, Shanghai, China), transferred to polyvinylidene fluoride (PVDF) membranes (Thermo Fisher, Waltham, MA, USA), and blocked with 5% non-fat milk for 2 h to block nonspecific binding. Thereafter, the membranes were incubated with primary antibodies at 4°C overnight, including AURKA and GPX4 [1:500; Cell Signaling Technology (CST), Danvers, MA, USA]; FTH1 (1:1,000; Affinity, Madison, WI, USA); xCT, FTL, and PTGS2 (1:1,000; Proteintech, Wuhan, China); PTGS2, ACSL4, SLC7A5, and PHKG2 (1:1,000; Sangon Biotech, Shanghai, China); and β-actin (1:2,000; CST, Danvers, MA, USA). Following washings, the membranes were incubated with horseradish peroxidase (HRP)-conjugated anti-mouse or anti-rabbit secondary antibodies (1:2,000; CST, Danvers, MA, USA) for 1 h at room temperature. Subsequently, the protein signal was detected using a Gel Doc™ XR+ Gel Documentation System (Bio-Rad, Hercules, CA, USA) with enhanced chemiluminescence (ECL) reagents (Bio-Rad, Hercules, CA, USA).

### Determination of Malondialdehyde and Reduced Glutathione

The levels of malondialdehyde (MDA) and GSH were measured using the Microscale MDA Assay Kit and the Reduced GSH Assay Kit, respectively (both from Jiancheng Bioengineering, Nanjing, China).

### Determination of Intracellular Iron Concentration

According to the instructions of the manufacturer of the iron ion kit (Jiancheng Bioengineering, Nanjing, China), the cells were mixed with phosphate-buffered saline (PBS, 1:9) and were centrifuged at 4°C at 2,500 rpm for 10 min to obtain the supernatant. The supernatant was then mixed with the prepared buffer, centrifuged again, and the absorbance value at 520 nm measured to calculate the iron ion level.

### ROS Determination Assay

A549 cells with DCFH-DA (10 μM) were incubated at 37°C for 20 min, according to the instructions of the ROS detection kit manufacturer (Beyotime, Shanghai, China). Thereafter, the ROS level in the cells was examined using a fluorescence microscope (TCS SP8, Leica, Wetzlar, Germany).

### JC-1 Mitoscreen Assay

An appropriate amount of cells was collected and mixed with a JC-1 staining working solution and then incubated at 37°C for 20 min. The mixture was then centrifuged and washed twice. The fluorescence of the cells was imaged using a fluorescence microscope (TCS SP8, Leica, Wetzlar, Germany).

### *In Vivo* Xenograft Assay

BALB/c nude mice (4 weeks old) were maintained in a specific pathogen-free (SPF) environment. Animal welfare and the experimental procedures were in compliance with the National Institutes of Health Guidelines for the care and use of laboratory animals, and all procedures involving the mouse xenograft model were approved by the Ethics Review Committee of Nanjing University of Chinese Medicine. The luciferase-expressing A549 cell line with lentivirus was established to generate the orthotopic xenograft lung cancer model. A549 cells (2 × 10^7^ in 0.2 ml medium of a 1:1 mixture of RPMI 1640 and Matrigel 354248) were then injected into the right lung parenchyma of mice. Two weeks later, the mice were randomly divided into an OP-B-treatment group (2.5 mg/kg OP-B, *n* = 6) and a control group (treated with saline, *n* = 6). Treatment was administered to all mice *via* intraperitoneal (i.p.) injection (daily). The volume and the metastasis of tumors were determined by *in vivo* bioluminescence measurements (IVIS Spectrum, PerkinElmer, Waltham, MA, USA) after an i.p. injection of 200 μl d-Luciferin substrate (15 mg/ml in Dulbecco’s PBS; PerkinElmer, Waltham, MA, USA). On day 45 of i.p. injection, all tumors were collected and the following tests were carried out.

### Transmission Electron Microscope Assay

The model and the OP-B groups were selected for transmission electron microscopy (TEM) analysis in order to observe the mitochondrial ultrastructure of the A549 cells. Part of the lung tissues of mice was respectively fixed with 3% glutaraldehyde and 1% osmic acid for 2 h. The tissues were dehydrated through a graded ethanol series and then embedded, polymerized, repaired, and finally sliced. After staining with uranyl acetate and lead citrate, the sections were observed using a transmission electron microscope (JEM-1011).

### Immunofluorescence

Frozen sections were incubated at 4°C with pre-cooled acetone for 10 min, washed with PBS three times, and then blocked with 5% goat serum and 0.3% Triton X-100 in PBS for 1 h. Thereafter, the frozen sections underwent an overnight incubation with the primary antibodies AURKA (1:500; CST, Danvers, MA, USA) and GPX4 (1:200; Beyotime, Shanghai, China) in an antibody dilution buffer (ADB) [1× PBS/1% bovine serum albumin (BSA)/0.3% Triton X-100] at 4°C. Following washings, the frozen sections were incubated with a fluorochrome-conjugated anti-rabbit secondary antibody (1:1,000; CST, Danvers, MA, USA) and a fluorochrome-conjugated anti-mouse secondary antibody (1:400; ZSGB-Bio, Beijing, China) in ADB for 2 h at room temperature. Subsequently, the cells were stained with DAPI (1 μg/ml; CST, Danvers, MA, USA) for 5 min. Images were obtained under a fluorescence microscope (TCS SP8, Wetzlar, Germany, Leica).

Statistical Analysis

Student’s *t*-test was adopted to compare the gene expression between tumor tissues and adjacent non-tumor tissues or that of two groups. The chi-squared test was applied to compare the different proportions and clinicopathological characteristics. The OS between the high-risk and low-risk groups was assessed with the Kaplan–Meier (KM) survival analysis and the two-sided log-rank test. The independent prognostic factors of OS were determined with univariate and multivariate Cox regression analyses. Two-way ANOVA was selected when repeated measures were compared. Data from the experiments are presented as the mean ± standard deviation (SD). If not specified, *p* < 0.05 was considered statistically significant, and all of the *p*-values examined for statistical significance were two-tailed. All statistical analyses were performed using *R* software (version 4.0.3) and GraphPad Prism 8.0 software.

## Results

### Identification of Prognostic Ferroptosis-Related Differentially Expressed Genes

**Figure 1:**
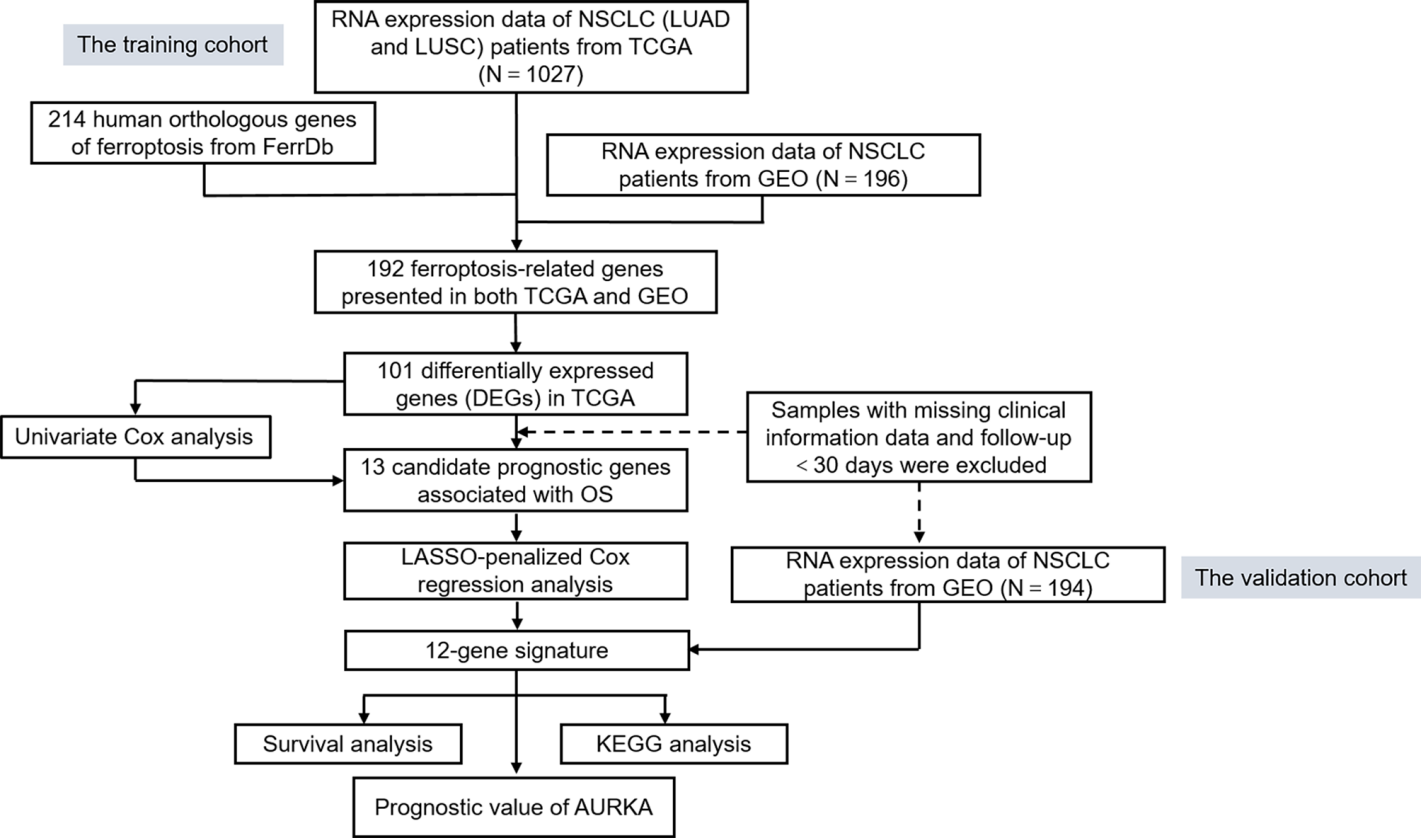
Flowchart of the bioinformatics analysis.

**Figure 2:**
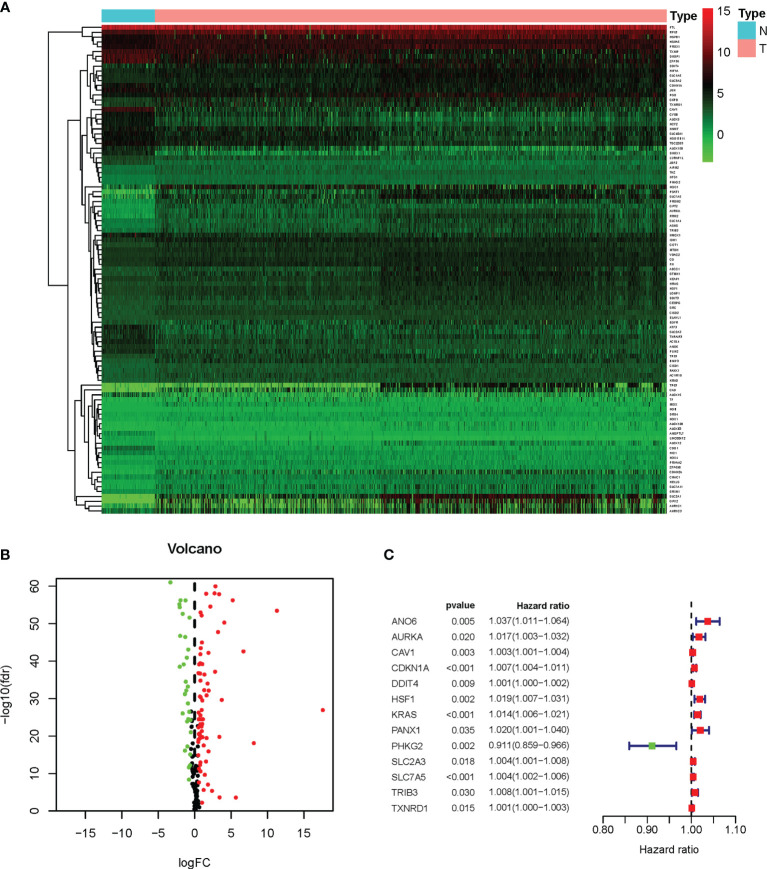
Survival-related differentially expressed ferroptosis genes. **(A)** Heat map of the differential expression of the ferroptosis genes in TCGA-LUAD and TCGA-LUSC datasets. *N*, normal; *T*, tumor. The expression intensity was based on the analysis of the gene expression level using R software. **(B)** Volcano plot of the gene expression data. *Red dots* denote upregulation, *green dots* show downregulation, and *black dots* denote non-DEGs. *P* < 0.05 was considered as significant. **(C)** Forest plot of the univariate Cox regression analysis showing the survival-related DEGs in the TCGA-LUAD and TCGA-LUSC datasets. *DEGs*, differentially expressed genes; *TCGA*, The Cancer Genome Atlas; *LUAD*, lung adenocarcinoma; *LUSC*, lung squamous cell carcinoma.

To systematically introduce this study, the flowchart of the bioinformatics analysis is shown in **Figure 1**. The TCGA training cohort, consisting of the TCGA-LUAD and TCGA-LUSC datasets, contained a total of 1,037 cancerous tissue samples and 108 adjacent normal tissue samples. The validation cohort, the GSE37745 microarray dataset, contained 196 samples. The vast majority of the ferroptosis-related genes (192/214, 89.7%) were present in the above two datasets. A total of 101 ferroptosis-associated DEGs in NSCLC were screened using the “limma” package in *R*, with |logFC| > 0.5 and FDR < 0.05. A heat map was drawn to show the hierarchical cluster analysis of the 101 ferroptosis-related DEGs in tumor and normal tissues (**Figure 2A**), and a volcano plot was used to visualize the DEGs (**Figure 2B**). Cases with missing clinical information data and follow-up of less than 30 days were excluded. Subsequently, 956 patients from the TCGA-LUAD and TCGA-LUSC cohorts and 194 patients from GSE37745 were extracted for prognostic analysis. A total of 13 candidate prognostic genes significantly associated with OS were retained by univariate Cox regression analysis (*p* < 0.01; **Figure 2C**).

### Construction and Validation of the Prognostic Signature

**Figure 3:**
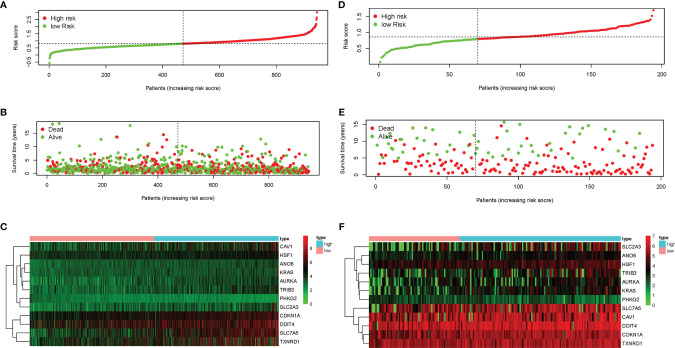
Characteristics of the prognostic genes in the gene signature. **(A, B, D, E)** Distribution of risk scores **(A)** and the patient survival time and non-small cell lung cancer (NSCLC) status **(B)** in The Cancer Genome Atlas (TCGA) and the Gene Expression Omnibus (GEO) **(D, E)**. The *black dotted line* is the optimal cutoff value used to classify patients into the low-risk and high-risk groups. **(C, F)** Heat map of the ferroptosis-related gene expression profiles in the prognostic features of NSCLC in TCGA **(C)** and GEO **(F)**. “*High*” represents the high-risk group and “*low*” the low-risk group in the heat map.

LASSO-penalized Cox regression analysis was performed on the prognostic ferroptosis gene markers in TCGA and then verified by combining the datasets of the ferroptosis genes and the survival time in GEO. After random simulations and cross-validation for 1,000 times to minimize the risk of overfitting, 12 genes significantly associated with OS were retained in the prognostic model out of the 13 candidate prognostic genes analyzed using univariate Cox regression. The coefficients of the 12-gene signature are shown in **Table 1**. We obtained signature-based risk scores by calculating the gene expression levels and the regression coefficients. According to the median risk scores, patients were stratified into a high-risk group (*n* = 472) or a low-risk group (*n* = 474) in the TCGA cohort (**Figure 3A**). As shown in the distribution of the survival time and disease status of patients, those with a 5-year survival time in the low-risk group, especially those with a 10-year survival time, were more than those in the high-risk group (**Figure 3B**). The heat map showed that, as the risk scores increased, the expression levels of 11 genes also increased, with *PHKG2* showing the opposite trend (**Figure 3C**). To verify the rigor of the model constructed from the TCGA-LUAD and TCGA-LUSC cohorts, the microarray dataset GSE37745 was employed for subsequent analysis. As previously mentioned, patients were categorized into high-risk and low-risk groups based on the median risk scores of the TCGA training cohort (**Figure 3D**). Similarly, the mortality rate of the patients in the high-risk group was higher than that in the low-risk group (**Figure 3E**). Except for *PKHG2*, all the remaining genes were verified as high-risk genes in the GSE37745 dataset (**Figure 3F**). *P* < 0.05 was regarded as statistically significant.

**Table 1:** Genes included in the prognostic gene signature.

Gene symbol	Full name	Coefficient
*ANO6*	Anoctamin 6	0.012655
*AURKA*	Aurora kinase A	0.002017
*CAV1*	Caveolin 1	0.00112
*CDKN1A*	Cyclin-dependent kinase inhibitor 1A	0.005417
*DDIT4*	DNA damage inducible transcript 4	0.000405
*HSF1*	Heat shock transcription factor 1	0.018154
*KRAS*	KRAS proto-oncogene, GTPase	0.011042
*PHKG2*	Phosphorylase kinase catalytic subunit gamma 2	−0.05625
*SLC2A3*	Solute carrier family 2 member 3	0.002083
*SLC7A5*	Solute carrier family 7 member 5	0.001001
*TRIB3*	Tribbles pseudokinase 3	0.00264
*TXNRD1*	Thioredoxin reductase 1	0.001341

### Independent Prognostic Value of the 12-Gene Signature

**Figure 4:**
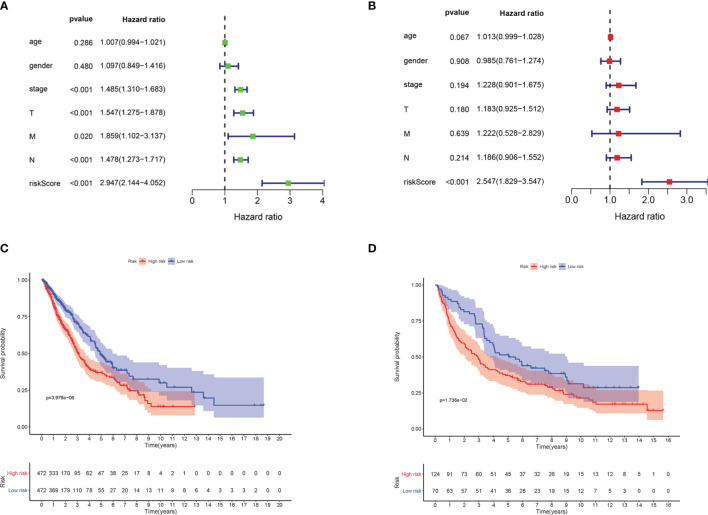
The ferroptosis-related gene markers were significantly associated with survival in non-small cell lung cancer (NSCLC). **(A)** Univariate Cox regression analysis. Forest map of the relationship between the risk factors and survival in the TCGA-LUAD and TCGA-LUSC datasets. **(B)** Multiple Cox regression analysis. The ferroptosis-related gene signatures are independent predictors in TCGA-LUAD and TCGA-LUSC. **(C)** Kaplan–Meier analysis of patients in the TCGA-LUAD and TCGA-LUSC datasets stratified by median risk. High-risk scores were associated with general poor survival in TCGA-LUAD and TCGA-LUSC. **(D)** GEO database survival analysis verification. *T*, tumor; *N*, node; *M*, metastasis; *TCGA*, The Cancer Genome Atlas; *LUAD*, lung adenocarcinoma; *LUSC*, lung squamous cell carcinoma; GEO, Gene Expression Omnibus.

Univariate and multivariate Cox regression analyses were implemented to examine whether the risk score is an independent predictive factor of OS (**Table 2**). In the TCGA-LUAD and TCGA-LUSC cohorts, the risk score was significantly associated with OS in the univariate regression analysis [hazard ratio (HR) = 2.947, 95% CI = 2.144–4.052, *p* < 0.001] (**Figure 4A**). Multivariate Cox regression analysis confirmed that the risk score was an independent predictor after revising for other confounding factors (HR = 2.547, 95% CI = 1.829–3.547, *p* < 0.001) (**Figure 4B**). The KM survival curves showed that the survival time of the patients in the low-risk group was significantly longer than that of patients in the high-risk group (*p* < 0.001; **Figure 4C**). Furthermore, the survival curve in the GEO cohort confirmed that patients in the low-risk group have better OS compared to their high-risk counterparts (*p* < 0.05; **Figure 4D**).

**Table 2:** Univariate and multivariate Cox regression analyses of the contribution of various potential prognostic factors to survival.

	Univariate Cox regression			Multiple Cox regression		
	HR	95% CI	*p*-value	HR	95% CI	*p*-value
TCGA						
Age (years)	1.007	0.994–1.021	0.286	1.013	0.999–1.028	0.067
Gender	1.097	0.849–1.416	0.480	0.985	0.761–1.274	0.908
Stage	1.485	1.310–1.683	<0.001	1.228	0.901–1.675	0.194
T	1.547	1.275–1.878	<0.001	1.183	0.925–1.512	0.180
M	1.859	1.102–3.137	0.020	1.222	0.528–2.829	0.639
N	1.478	1.273–1.717	<0.001	1.186	0.906–1.552	0.214
Risk score	2.947	2.144–4.052	<0.001	2.547	1.829–3.547	<0.001

TCGA, The Cancer Genome Atlas; T, tumor; N, node; M, metastasis.

### Kyoto Encyclopedia of Genes and Genomes Pathways on Gene Set Enrichment Analysis

**Figure 5:**
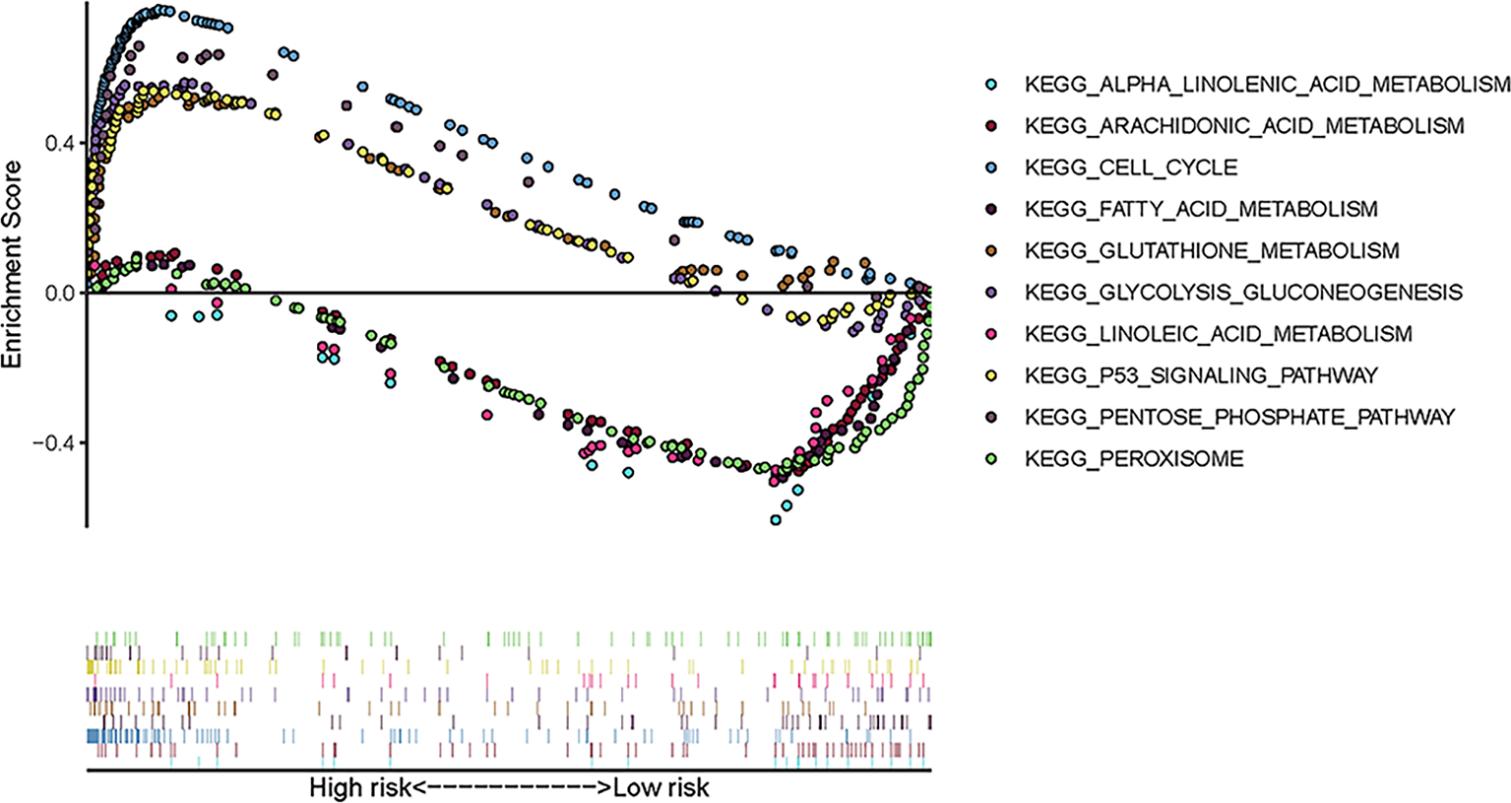
Ten representative enriched KEGG pathways by GSEA. Each group includes five KEGG pathways. The related parameters for the pathways are given in **Table 3**. *GSEA*, gene set enrichment analysis; *KEGG*, Kyoto Encyclopedia of Genes and Genomes.

To explore the enriched pathways that were significantly correlated with the risk score, we performed KEGG pathway analysis on the ferroptosis-related prognostic genes. The results of KEGG analysis showed that the DEGs were mainly enriched in several ferroptosis-associated pathways. A total of 10 signaling pathways are shown here. Five signaling pathways—cell cycle, p53 signaling, pentose phosphate, glycolysis–gluconeogenesis, and glutathione metabolism pathways—were enriched in patients in the high-risk group; the other five pathways—alpha linolenic acid metabolism, arachidonic acid metabolism, linoleic acid metabolism, peroxisome, and fatty acid metabolism—were enriched in patients in the low-risk group (*p* < 0.05; **Figure 5**). The relevant parameters for the KEGG pathways are listed in **Table 3**. The above results suggest the mechanism of ferroptosis in LUAD and LUSC.

**Table 3:** Relevant parameters of the KEGG pathways in **Figure 5**.

	Size	ES	NES	*p*-value	FDR-q
High risk					
KEGG_CELL_CYCLE	125	0.75	2.42	<0.001	<0.001
KEGG_P53_SIGNALING_PATHWAY	68	0.54	2.05	0.002	0.022
KEGG_PENTOSE_PHOSPHATE_PATHWAY	27	0.66	2.00	0.004	0.030
KEGG_GLYCOLYSIS_GLUCONEOGENESIS	62	0.56	1.93	0.002	0.033
KEGG_GLUTATHIONE_METABOLISM	49	0.521	1.65	0.049	0.067
Low risk					
KEGG_ALPHA_LINOLENIC_ACID_METABOLISM	19	−0.65	−2.01	<0.001	0.068
KEGG_ARACHIDONIC_ACID_METABOLISM	58	−0.49	−1.84	0.004	0.084
KEGG_LINOLEIC_ACID_METABOLISM	29	−0.54	−1.76	0.002	0.126
KEGG_PEROXISOME	78	−0.48	−1.74	0.031	0.116
KEGG_FATTY_ACID_METABOLISM	42	−0.512	−1.67	0.033	0.141

KEGG, Kyoto Encyclopedia of Genes and Genomes; ES, enrichment score; NES, normalized enrichment score.

### *AURKA* Expression Is Significantly Increased in NSCLC

**Figure 6:**
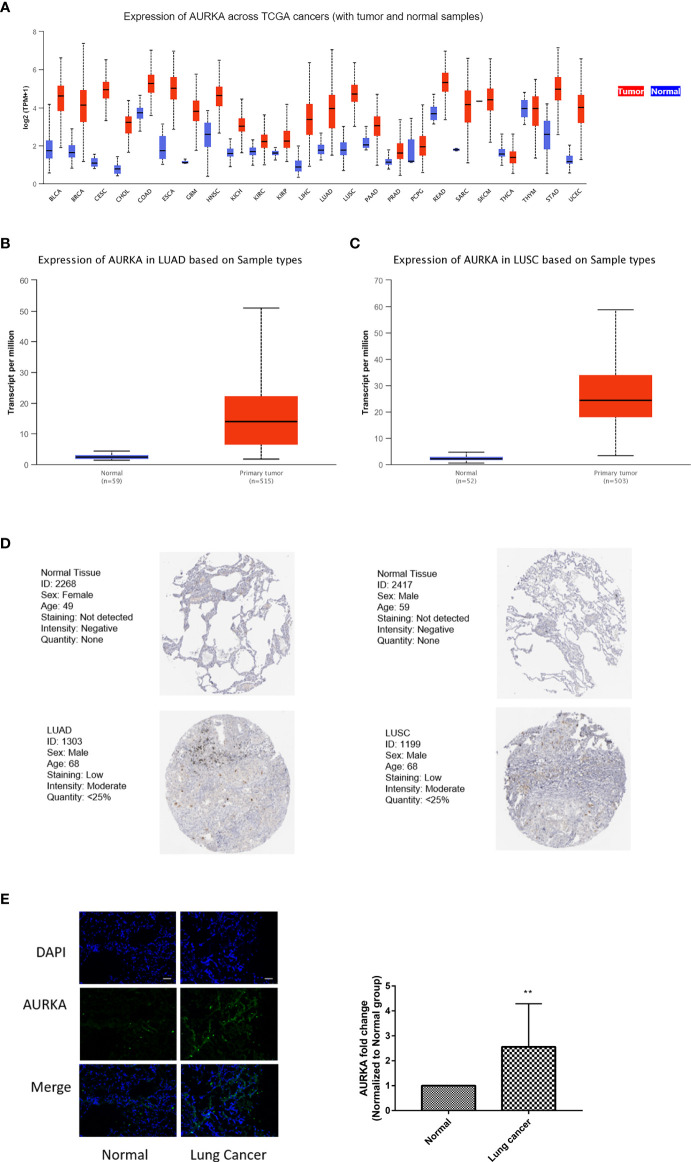
*AURKA* expression was significantly increased in non-small cell lung cancer (NSCLC). **(A)** Comparison of the expression of *AURKA* between tumor and normal samples. Differences of the expression of *AURKA* between tumor tissues and adjacent normal tissues in lung adenocarcinoma (LUAD) **(B)** and lung squamous cell carcinoma (LUSC) **(C)** from The Cancer Genome Atlas (TCGA). **(D)** Immunohistochemistry (IHC) results from the Human Protein Atlas (HPA) showing the protein levels of *AURKA* in normal and tumor tissues. **(E)** Immunofluorescence staining of *AURKA* expression in 11 pairs of tissue samples from patients with lung cancer. *Bars* and *error bars* indicate the mean ± SD. **p < 0.01.

*AURKA* has attracted great attention among those in the 12-gene signature as it is a classical cell cycle-regulated protein kinase, and little is known about its role in ferroptosis. Analysis of the TCGA dataset revealed that *AURKA* was abnormally expressed in 24 tumors and involved in tumor progression. Compared with that in corresponding normal tissues, the expression of *AURKA* mRNA was significantly increased in almost all tumors, except in thymoma (**Figure 6A**). Similarly, *AURKA* mRNA was also highly expressed in NSCLC, including LUAD and LUSC (**Figures 6B, C** **)**. At the same time, the HPA database was used to analyze the differences in the protein expression of *AURKA* in normal and lung cancer tissues. As shown in **Figure 6D**, the expression of *AURKA* in LUAD or LUSC was higher than that in normal tissues. We collected a total of 11 pairs of tissue samples from patients with NSCLC and examined the expression of *AURKA* by immunofluorescence, which further confirmed the above conclusion (**Figure 6E**). Summarily, *AURKA* is highly expressed in NSCLC.

### *AURKA* Is a Significant Prognostic Biomarker in NSCLC

**Figure 7:**
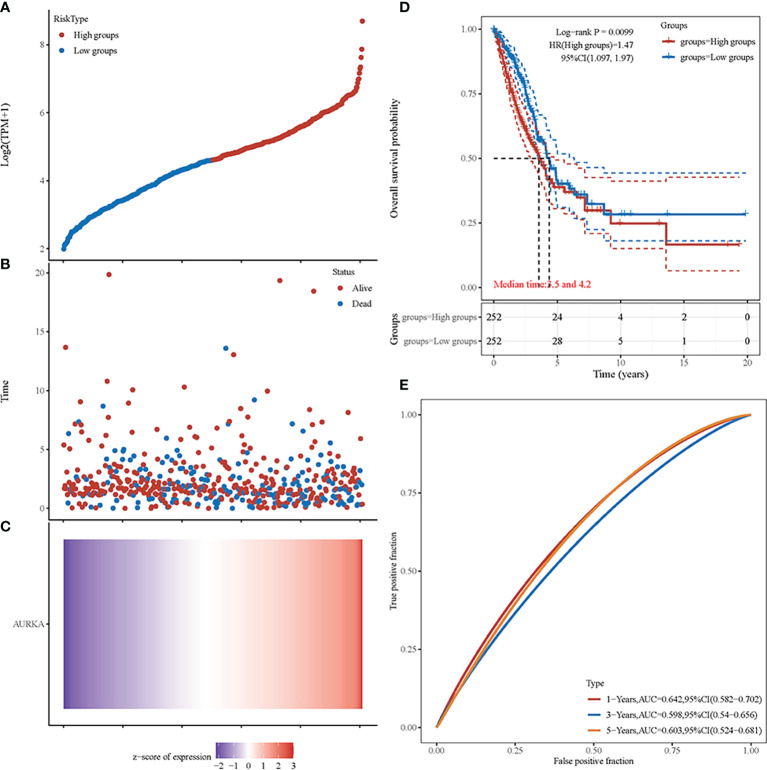
Prognostic analysis of *AURKA* in non-small cell lung cancer (NSCLC). **(A)** Curve of the risk scores. **(B)** Survival time and status of patients. More dead patients correspond to the higher risk scores. **(C)** Heat map of the expression profiles of *AURKA*. **(D)** Kaplan–Meier survival analysis of *AURKA*. **(E)** Time-dependent receiver operating characteristic (ROC) analysis.

We performed an independent analysis to further determine the prognostic value of *AURKA*. From the TCGA dataset (https://portal.gdc.com), the original counts and the corresponding clinical information of the RNA-seq data (level 3) of NSCLC were obtained. Patients were classified into a high-risk group (*n* = 252) or a low-risk group (*n* = 252) according to the median risk score of the *AURKA* gene signature (**Figure 7A**). **Figure 7B** shows the survival status of all patients. Patients in the low-risk group showed better survival than those in the high-risk group. The heat map reconfirmed that *AURKA* is a high-risk gene (**Figure 7C**). The KM survival curve showed that the OS of the patients in the high-risk group was worse than that of patients in the low-risk group (**Figure 7D**). Moreover, in the time-dependent receiver operating characteristic curve (ROC) analysis, the *AURKA* prognostic signature showed meaningful area under the curve (AUC) values. The AUC values were as follows: 1 year, 0.642; 2 years, 0.598; and 3 years, 0.603 (**Figure 7E**).

### Inhibition of *AURKA* Promotes Ferroptosis in NSCLC Cells

**Figure 8:**
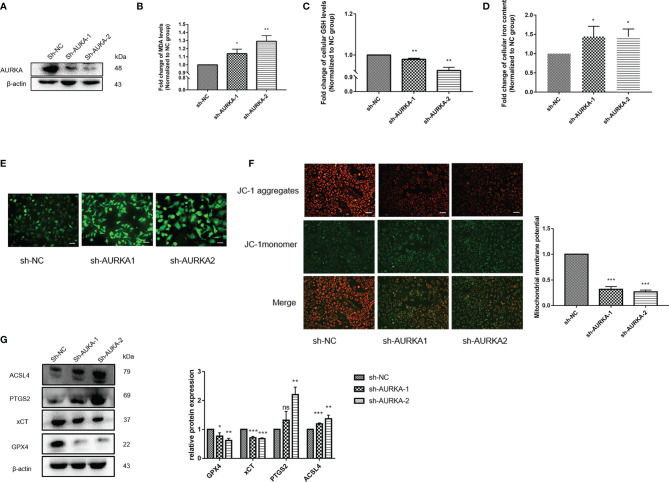
Inhibition of *AURKA* promoted ferroptosis in non-small cell lung cancer (NSCLC) cells. **(A)** Western blot analysis of the expression level of *AURKA* in A549 cells transfected with the short hairpin RNA plasmids sh-AURKA-1 and sh-AURKA-2. **(B)** Level of malondialdehyde (MDA) in A549 cells. **(C)** Level of glutathione (GSH) in A549 cells. **(D)** Concentration of intracellular iron. **(E)** Reactive oxygen species (ROS) determination assay. Magnification, ×200. *Scale bar*, 50 μm. **(F)** Mitochondrial membrane potential (MMP) assay. Magnification, ×200. *Scale bar*, 50 μm. **(G)** Western blot of the levels of the ferroptosis-related proteins GPX4, xCT, PTGS2, and ACSl4. The assay was repeated three times. *Data bars* and *error bars* indicate the mean ± SD. *p < 0.05, **p < 0.01, ***p < 0.001. *ns*, no significance.

Ferroptosis is an iron-dependent non-apoptotic cell death that is closely related to the survival of NSCLC cells. To clarify the effect of *AURKA* on ferroptosis, we first analyzed the RNA expression of *AURKA* across different cell lines using the HPA database. As shown in **Supplementary Figure S1A**, the relative RNA expression level of *AURKA* in LUAD A549 cells was higher than that in normal human bronchial epithelial HBEC3-KT cells. On the other hand, we obtained the expression of *AURKA* in different LUAD cell lines through the CCLE database. The cell line expression matrix showed that *AURKA* was highly expressed in A549 cells and was selected for subsequent experimental analysis (**Supplementary Figure S1B**). Several research teams have reported that the expression of *AURKA* in the NSCLC cell line A549 was higher than that in the normal lung cell line HBE (31, 32). To evaluate the biological role of *AURKA* in ferroptosis in NSCLC, we chose plasmid-mediated inhibition to exogenously regulate the expression of *AURKA* in A549 cells. The Western blot results confirmed that the expression of *AURKA* was significantly downregulated after transfection with sh-AURKA-1 or sh-AURKA-2 compared with the control group (**Figure 8A**). We also found that, compared with the sh-NC group, the MDA levels markedly increased after *AURKA* silencing (**Figure 8B**) and the GSH levels decreased (**Figure 8C**). Moreover, we observed that the intracellular iron levels (**Figure 8D**) and ROS production (**Figure 8E**) increased after *AURKA* silencing, while the mitochondrial membrane potential (MMP) was significantly reduced (**Figure 8F**). Thereafter, we determined the ferroptosis-related proteins GPX4, xCT, prostaglandin-endoperoxide synthase 2 (PTGS2), and acyl-CoA synthetase long-chain family member 4 (ACSL4) with Western blot. Compared with the sh-NC group, the protein expression of the ferroptosis-inhibiting proteins GPX4 and xCT was downregulated after *AURKA* knockdown. On the contrary, the protein expression of the ferroptosis-promoting proteins PTGS2 and ACSL4 was notably upregulated (**Figure 8G**). These results suggested that the inhibition of *AURKA* promoted ferroptosis in NSCLC cells.

### OP-B Induces Ferroptosis in NSCLC Cells

**Figure 9:**
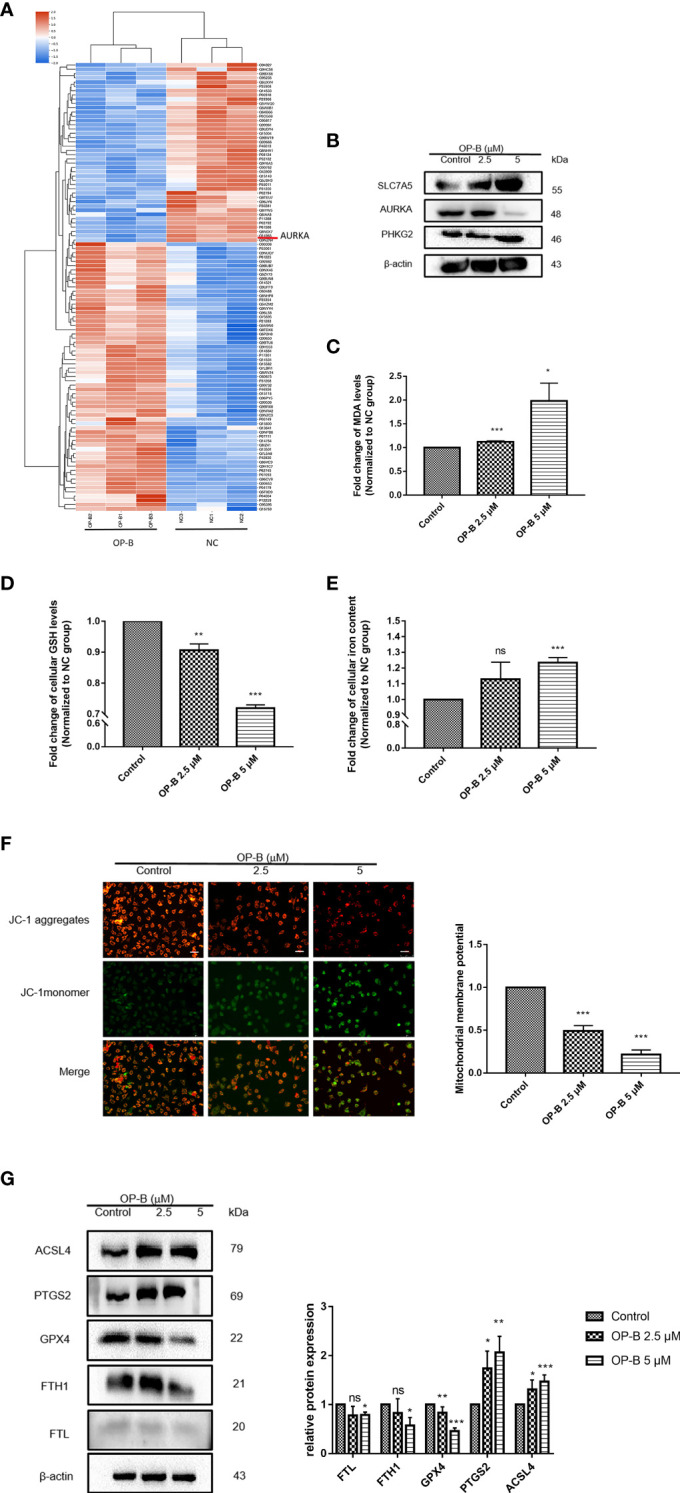
Ophiopogonin B (OP-B) induced ferroptosis in non-small cell lung cancer (NSCLC) cells. **(A)** Heat map of the differential expressions of the screened protein sequences in A549 cells treated or not treated with OP-B for 24 h. **(B)** Protein expression of *SLC7A5*, *PHKG2*, and *AURKA* in A549 cells after treatment with OP-B for 24 h. **(C)** For the malondialdehyde (MDA) assay, A549 cells were treated with dimethyl sulfoxide (DMSO) or OP-B for 24 h. **(D)** For glutathione (GSH) level, A549 cells were treated with DMSO or OP-B for 24 h. **(E)** For the detection of the intracellular iron concentration, A549 cells were treated with DMSO or OP-B for 24 h. **(F)** The mitochondrial membrane potential (MMP) assay was performed after treatment of A549 cells with DMSO or OP-B for 24 h. Magnification, ×200. *Scale bar*, 50 μm. **(G)** Western blot of the levels of the ferroptosis-related proteins FTL, FTH1, GPX4, PTGS2, and ACSl4 after treatment of A549 cells with DMSO or OP-B for 24 h. The assay was repeated three times. *Data bars* and *error bars* indicate the mean ± SD. *p < 0.05, **p < 0.01, ***p < 0.001. *ns*, no significance.

To further explore the molecular mechanism of OP-B against NSCLC, we performed proteomic sequencing of A549 cells administered OP-B. Results of the analysis of the protein GO terms showed that OP-B influenced cell ferric iron binding compared with the NC group (**Supplementary Figure S2A**). KEGG pathway analysis also suggested that OP-B affected the genes of the ferroptosis pathway (**Supplementary Figure S2B**). Protein cluster analysis showed that the expression of AURKA, ferritin heavy chain 1 (FTH1), and ferritin light chain (FTL) significantly decreased, while that of ACSL4 increased in OP-B-treated NSCLC cells (**Figure 9A**). Because a 12-gene signature prognostic model in NSCLC had been constructed previously, we detected the expression of these genes in A549 cells under OP-B treatment. The results showed that the expression of *SLC7A5* and *PHKG2* increased, while that of *AURKA* was dramatically decreased by OP-B (**Figure 9B**). To further determine whether OP-B induced ferroptosis in A549 cells, we examined MDA, GSH, and MMP, the key factors of ferroptosis, and found that the level of MDA increased in a dose-dependent manner, while that of GSH notably decreased in A549 cells that received OP-B treatment (**Figures 9C, D** **)**. On the other hand, the level of intracellular iron increased under 5 μM OP-B treatment (**Figure 9E**), and MMP was also significantly reduced (**Figure 9F**). Moreover, the results of Western blot showed that the ferroptosis negative regulatory proteins FTL, FTH1, and GPX4 were downregulated by 5 μM OP-B treatment, while the protein expression of the ferroptosis positive regulatory proteins PTGS2 and ACSL4 was upregulated (**Figure 9G**).

Overall, the *in vitro* experiments provided support for OP-B being able to prevent NSCLC progression by inducing ferroptosis.

### OP-B Induces Ferroptosis *In Vivo*

**Figure 10:**
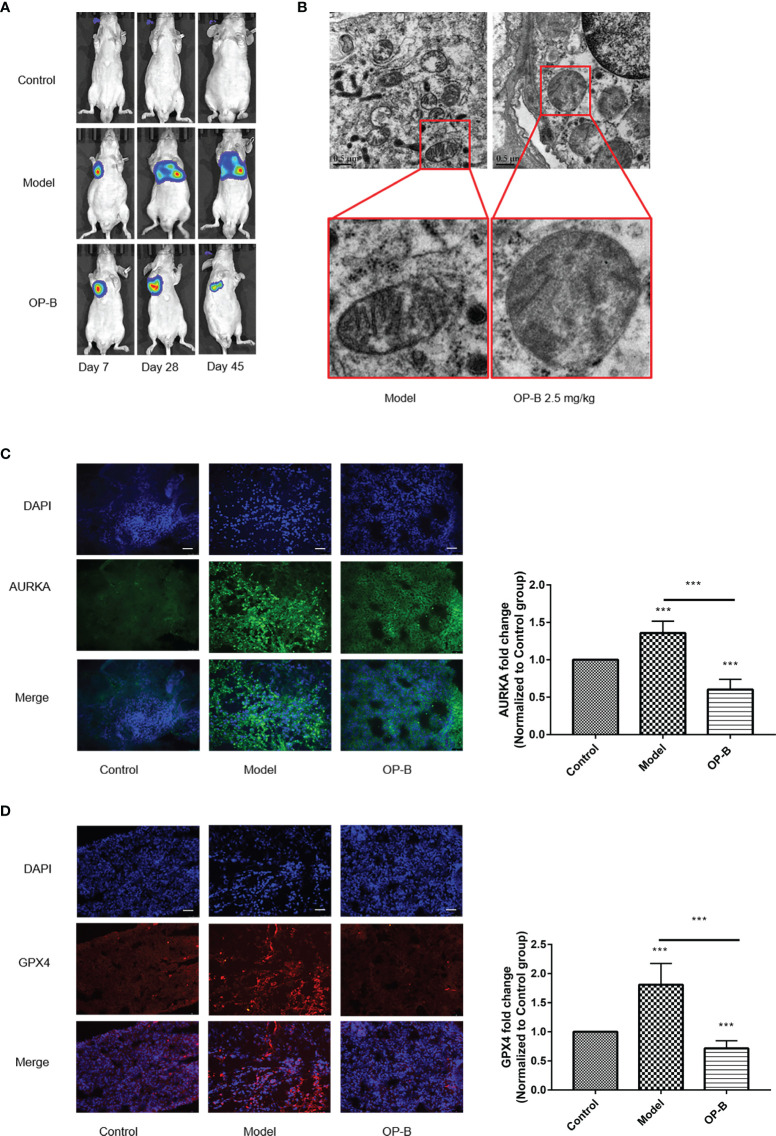
Ophiopogonin B (OP-B) induced ferroptosis *in vivo*. **(A)** Bioluminescence imaging of the OP-B-administered groups with right lung parenchyma injection of luciferase-labeled A549 cells. **(B)** Transmission electron microscopy images of the model group and the OP-B-administered group (2.5 mg/kg) in nude mice. **(C)** Immunofluorescence staining of the expression of *AURKA* in the model group and OP-B-treated group in nude mice. **(D)** Immunofluorescence staining of the expression of GPX4 in the model group and OP-B-treated group in nude mice. *Data bars* and *error bars* indicate the mean ± SD. ***p < 0.001.

In previous studies, the extensive antitumor effect of OP-B has been reported *in vivo* and *in vitro*. In order to reconfirm the anti-NSCLC effect of OP-B, we firstly injected A549 cells into the lung parenchyma of BALA/c nude mice and then administered OP-B treatment. The bioluminescence images showed that the proliferation and metastasis of NSCLC cells tracked by luciferase were significantly inhibited at the OP-B concentration of 2.5 mg/kg (**Figure 10A**). The ultrastructure of the lung cancer tissues in the model group and in the 2.5-mg/kg OP-B group was detected by TEM. The TEM images showed that, compared with the control group, OP-B administration promoted the mitochondria to become rounder; increased the mitochondrial membrane density; and decreased, damaged, and vanished the mitochondrial cristae (**Figure 10B**). Immunofluorescence of the frozen sections showed that the fluorescence intensity of the ferroptosis suppressor genes *AURKA* and *GPX4* was reduced after OP-B treatment in the test groups (**Figures 10C, D** **)**.

Together, the above experimental results show that OP-B significantly induced ferroptosis in tumor tissues in nude mice.

### Induction of Ferroptosis by OP-B in NSCLC Cells Through the Downregulation of *AURKA* Expression

**Figure 11:**
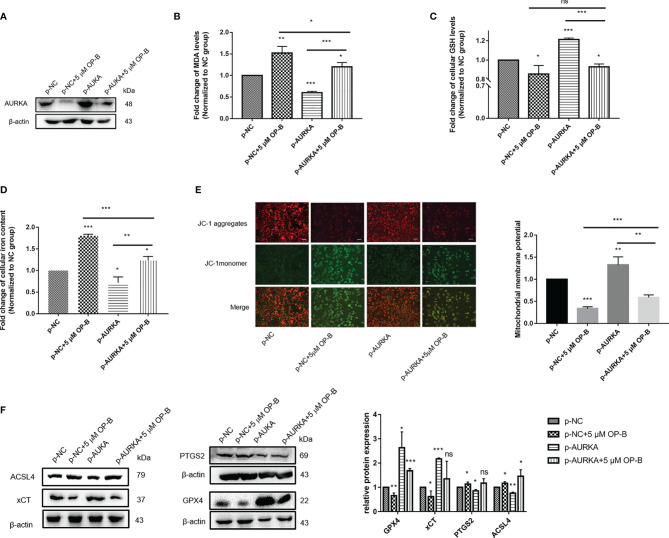
Induction of ferroptosis by ophiopogonin B (OP-B) in non-small cell lung cancer (NSCLC) cells through the downregulation of *AURKA* expression. **(A)** The expression of *AURKA* was determined using Western blot. **(B)** Level of malondialdehyde (MDA). **(C)** Level of glutathione (GSH). **(D)** Concentration of intracellular iron. **(E)** Mitochondrial membrane potential (MMP) assay. Magnification, ×200. *Scale bar*, 50 μm. **(F)** Western blot of the levels of the ferroptosis-related proteins GPX4, xCT, PTGS2, and ACSL4. The Western blot experiments for all proteins were repeated three times, except that for ACSL4 (repeated twice). *Data bars* and *error bars* indicate the mean ± SD. *p < 0.05, **p < 0.01, ***p < 0.001. *ns*, no significance.

To further confirm that the promotion of ferroptosis by OP-B was achieved by regulating the expression of *AURKA*, we attempted to overexpress *AURKA* in A549 cells in order to observe the ferroptosis-associated biological indexes of OP-B combined with *AURKA* overexpression. We used plasmid-mediated overexpression to upregulate the expression of *AURKA*. The Western blot results showed that the expression of *AURKA* was effectively upregulated in A549 cells transfected with p-AURKA compared with the control cells. Consistent with previous results, *AURKA* was significantly downregulated after OP-B treatment, but partially reversed by *AURKA* overexpression (**Figure 11A**). On the other hand, after overexpressing *AURKA*, the levels of MDA (**Figure 11B**) and intracellular iron (**Figure 11D**) decreased significantly, while the levels of GSH (**Figure 11C**) and MMP (**Figure 11E**) increased (all *p* < 0.05). These results suggested that the overexpression of *AURKA* inhibited ferroptosis in A549 cells. In contrast, OP-B promoted ferroptosis, but the inhibition of ferroptosis by *AURKA* overexpression partially reversed the effect of OP-B. Subsequently, we examined the expression of ferroptosis-associated proteins, which showed results contrary to the effect of OP-B administration, where the overexpression of *AURKA* upregulated the protein expression of GPX4 and xCT, but downregulated that of PTGS2 and ACSL4 (**Figure 11F**). In addition, the downregulation of GPX4 and xCT caused by OP-B was partially restored by *AURKA* overexpression, while the upregulation of PTGS2 and ACSL4 was partially reversed. All these results further indicated that OP-B promotes ferroptosis in NSCLC cells by downregulating the expression of *AURKA*.

## Discussion

Ferroptosis, a form of iron-dependent programmed cell death, differs from other types of cell death, such as apoptosis, pyrolysis, and autophagy (33) and has great potential for clinical practice in tumors. Sorafenib, for instance, is widely applied for the treatment of advanced liver cancer, and it induces ferroptosis by inhibiting system xc^−^ in order to enhance cytotoxicity and to inhibit the proliferation of hepatoma cells (34). Lin et al. revealed that dihydroartemisinin (DHA) exhibits specific antitumor activity in head and neck cancer *via* inducing ferroptosis in carcinoma cells (35). Artesunate (ART) has been proven to accelerate intracellular ROS accumulation and specifically induce ferroptosis in pancreatic ductal adenocarcinoma (PDAC) cell lines (36).

Compared with other tissues, the lung is exposed to higher oxygen concentrations, which greatly increases its risk of oxidative stress (20). Theoretically, ferroptosis is more likely to occur in lung cancer, but it is not established. Several pieces of evidence have demonstrated that ferroptosis inhibition is ubiquitous in lung cancer cells. In patients with NSCLC, the expression of ferritin is higher in tumor tissues than that in normal tissues, and H-ferritin prevents the ferroptosis of cancer cells by controlling the Fenton reaction (37, 38). Lymphoid-specific helicase (LSH) acts as an oncogene in lung cancer that inhibits ferroptosis by activating lipid metabolism (39). *STYK1* shows carcinogenicity by increasing the expression of GPX4 and then controlling ferroptosis in lung cancer cells (19).

Meanwhile, according to these ferroptosis molecular targets, numerous new antitumor mechanisms have been correspondingly developed. It was found that cisplatin causes ferroptosis in A549 cells through the depletion of reduced GSH and the inactivation of GPX4. Further studies showed that the combination therapy of cisplatin and erastin has a remarkable synergistic effect on its antineoplastic activity (40). Gai et al. observed that APAP increases the sensitivity of erastin-induced ferroptosis by regulating the Nrf2/heme oxygenase 1 signaling pathway in NSCLC (41). All of these studies have shown that a more in-depth understanding of the molecular mechanism of ferroptosis is conducive to developing ferroptosis target therapy and improving the antitumor therapeutic effect in NSCLC.

In this study, we used the TCGA-LUAD and TCGA-LUSC datasets as the training sample and the GSE37745 dataset as the test sample to verify our prediction model. A total of 101 ferroptosis-related genes with significantly different expressions were screened out. Thirteen survival-related genes in NSCLC were revealed using univariate regression analysis, and LASSO regression analysis carried out to establish a 12-gene prognostic model. Both univariate and multivariate Cox regression analyses explored whether the model was an independent asymptotic factor. According to the characteristics of the 12 genes, the risk score of each patient was obtained by calculating the mRNA expression level and the risk coefficient of the selected genes. After verification, the risk score could be used as an independent prognostic indicator. The survival curves from the TCGA and GEO databases also demonstrated that patients in the high-risk group were associated with poorer prognosis, indicating that our study is meaningful. The prognostic model proposed in this study consisted of 12 ferroptosis-related genes (*ANO6*, *AURKA*, *CAV1*, *CDKN1A*, *DDIT4*, *HSF1*, *KRAS*, *PHKG2*, *SLC2A3*, *SLC7A5*, *TRIB3*, and *TXNRD1*). These genes could be approximately divided into four categories: driver genes (*ANO6*, *KRAS*, and *PHKG2*), suppressor genes (*HSF1*, *CAV1*, and *CDKN1A*), and marker genes (*AURKA*, *DDIT4 SLC2A3*, *SLC7A5*, *TRIB3*, and *TXNRD1*). Among those in the 12-gene signature, *AURKA*, as a ferroptosis predictive factor, has strongly attracted our attention. *AURKA* is a kinase that regulates the cell cycle, and it appears to be involved in microtubule formation and spindle pole stabilization during chromosome separation and plays a role in the occurrence and development of tumors. Inhibition of *AURKA* will inhibit GPX4 and induce ferroptotic cell death (42). We performed an independent prognostic value analysis of *AURKA*. The KM survival analysis and the ROC curves showed the great prognostic value of *AURKA* as a ferroptosis-related gene signature.

Although the 12-gene signature was confirmed in different databases, this study still has limitations. The ferroptosis risk predictive genes determined in this study need to be verified in more *in vivo* and *in vitro* experiments. Particularly, *AURKA*, as a classical oncogene, has been widely studied regarding the proliferation of cancer cells. However, there are too few studies on ferroptosis. Convincingly, Gomaa et al. reported that *AURKA* knockdown inhibited GPX4 and induced cell death, suggesting a link between *AURKA* and ferroptosis (42). However, there is still a lack of subsequent data elucidating the mechanism of *AURKA* in ferroptosis.

The main biological indicators of ferroptosis are the levels of MDA, GSH, intracellular iron, and intracellular ROS, as well as MMP, typically (43, 44). Because the significant high expression of *AURKA* in the LUAD cell line A549 has been fully verified, the A549 cell line was selected for subsequent *in vitro* experiments (31, 32). In this study, we knocked down *AURKA* in A549 cells by transfecting shRNA plasmid and found that the levels of MDA, intracellular iron, and ROS were notably increased, GSH was decreased, and MMP was reduced. Similar to other forms of programmed cell death, ferroptosis is regulated by various molecules and signals. GPX4 is the first confirmed negative regulatory protein of ferroptosis. It uses lipid ROS as a specific substrate and GSH as a reducing agent to remove lipid peroxide (45). Cystine/glutamate transporter (SLC7A11/xCT) is also a core negative regulatory protein of ferroptosis. It directly determines the content of reduced GSH by mediating the transport of cystine, a GSH precursor (33, 46). FTH1 and FTL can store intracellular free iron ions, and their reduced expression would lead to the increase of intracellular iron concentration and ferroptosis. Therefore, they are considered to be negative regulatory proteins of ferroptosis (47). ACSl4 is an important positive regulatory protein of ferroptosis that can promote the increase of lipid ROS and lead to ferroptosis (48). In addition, the transcriptional upregulation of PTGS2 is an important indicator of ferroptosis (45). This study found that the levels of the ferroptosis-inhibiting signaling pathway proteins GPX4 and xCT were notably downregulated in the sh-AURKA group, while those of the ferroptosis-promoting signaling pathway proteins PTGS2 and ACSL4 were upregulated. The above results indicate that inhibition of *AURKA* can promote ferroptosis in NSCLC cells and that *AURKA* acts as a ferroptosis suppressor.

OP-B is a bioactive component of Radix *O. japonicus*, which can effectively prevent the progression of NSCLC. In this study, the bioluminescence images once more confirmed the antitumor effect of OP-B *in vivo*. Analysis of the protein GO terms revealed that OP-B influenced the binding of intracellular ferric iron, and KEGG enrichment analysis showed that it was involved in the ferroptosis signaling pathway. The heat map of the differential proteins further indicated that OP-B downregulated the expression of the ferroptosis-inhibited proteins AURKA, GPX4, and FTL. In the *in vitro* experiments, 2.5 and 5 μM were selected as the effective doses of OP-B according to previous studies of relevant pharmacological mechanisms (49, 50). Further detection of the proteins by Western blot showed that OP-B can also regulate the expression of *SLC7A5* and *PHKG2*, which were included in the 12-gene signature we previously constructed. On the other hand, we detected the upregulation of positive regulatory proteins of ferroptosis, PTGS2 and ACSL4. All these suggest that OP-B can induce ferroptosis in NSCLC. Moreover, we confirmed that OP-B induced ferroptosis by examining the contents of MDA, GSH, and intracellular iron, as well as the MMP, *in vitro*. Furthermore, the density of the mitochondrial membrane was observed to be increased, while TEM displayed that the mitochondrial cristae decreased and disappeared after OP-B treatment *in vivo*. Consistent with previous results, immunofluorescence of frozen sections confirmed the downregulation of *AURKA* and GPX4 by OP-B treatment *in vivo*. Thus far, the induction of ferroptosis by OP-B has been fully verified.

Given that OP-B can downregulate the expression of *AURKA*, we propose to assume that the induction of ferroptosis is achieved by mediating *AURKA*. As expected, the ferroptosis-inducing effect caused by OP-B was partially suppressed in *AURKA*-overexpressed A549 cells, determined using the levels of MDA and GSH intracellular iron, as well as MMP. Similarly, the regulation of OP-B on the expression of ferroptosis-related proteins was partially reversed by *AURKA*. In conclusion, our study demonstrated that OP-B induces ferroptosis by regulating the expression of *AURKA*.

In summary, our study established a ferroptosis-related prognostic model. The 12-gene signature was predictive in the training and validation cohorts. Particularly, we confirmed for the first time that *AURKA* knockdown can promote ferroptosis in NSCLC and revealed that OP-B can induce ferroptosis in NSCLC by downregulating the expression of *AURKA*. Nevertheless, our research still needs to be improved. Firstly, the construction of the ferroptosis-related prognostic prediction model was based on bioinformatics analysis; future prospective studies need to be verified by animal experiments and clinical trials. Secondly, due to the current funding and cycle limitations, we cannot study all the genes included in the 12-gene signature. However, we will explore more ferroptosis targets in NSCLC in future research. In addition, *AURKA* knockdown promotes ferroptosis in NSCLC, but the specific molecular mechanism of OP-B-mediated *AURKA*-induced ferroptosis needs more *in vivo* experiments for verification.

## Conclusions

On the whole, our study established a ferroptosis-related prognostic model. The 12-gene signature was predictive in the training and validation cohorts. Moreover, we discovered, for the first time, the ferroptosis-inhibiting effect of *AURKA* and clarified that OP-B plays a ferroptosis-inducing role by regulating the expression of *AURKA*. With the advance of research on the mechanisms of ferroptosis induction in NSCLC, it will have great potential in the clinical treatment of NSCLC. Despite the significant anti-NSCLC effect of OP-B, its dosage form still needs to be improved due to its poor water solubility.

## Data Availability Statement

The datasets presented in this study can be found in online repositories. The names of the repository/repositories and accession number(s) can be found in the article/**Supplementary Material**.

## Ethics Statement

The studies involving human participants were reviewed and approved by the Ethics Committee on Human Research of Haian Hospital of Traditional Chinese Medicine (Nantong, China) (approval number: HZYLL2019-012). Patients/participants provided written informed consent to participate in this study. The animal study was reviewed and approved by the Institutional Animal Care and Use Committee of Nanjing University of Chinese Medicine (Nanjing, China) (approval number: 202012A006).

## Author Contributions

XZ and MC guided the experiment direction and revised the manuscript. LL designed the study, performed experiments, and wrote the manuscript. QG analyzed part of the data and animal experiments. JW performed the cell experiments. LG completed the frozen tissue section. YW, ZW, CH, and MH assisted in performing the cell experiments. YY, XFZ, and JF provided the project direction. FG, SZ, and ZL provided valuable advice. All authors contributed to the article and approved the submitted version.

## Funding

The present study was supported by the National Natural Science Foundation of China (no. 81503374), the Open Projects of the Discipline of Chinese Medicine of Nanjing University of Chinese Medicine Supported by the Subject of Academic Priority Discipline of Jiangsu Higher Education Institutions (PAPD) (grant no. ZYX03KF047), the Natural Science Foundation of Jiangsu Province (no. BK20181235), and the Natural Science Foundation of Nanjing University of Chinese Medicine (no. XZR2020084).

## Conflict of Interest

The authors declare that the research was conducted in the absence of any commercial or financial relationships that could be construed as a potential conflict of interest.

## Publisher’s Note

All claims expressed in this article are solely those of the authors and do not necessarily represent those of their affiliated organizations, or those of the publisher, the editors and the reviewers. Any product that may be evaluated in this article, or claim that may be made by its manufacturer, is not guaranteed or endorsed by the publisher.
