# Supplementary material for: Induction of Ferroptosis by Ophiopogonin-B Through Regulating the Gene Signature AURKA in NSCLC
Source: Front Oncol. 2022 Jun 28;12:833814. doi: 10.3389/fonc.2022.833814 (PMC9299951; doi:10.3389/fonc.2022.833814)
Supplement: Supplementary file 1 [file DataSheet_1.docx]

Supplementary Material

## Supplementary Figures

**
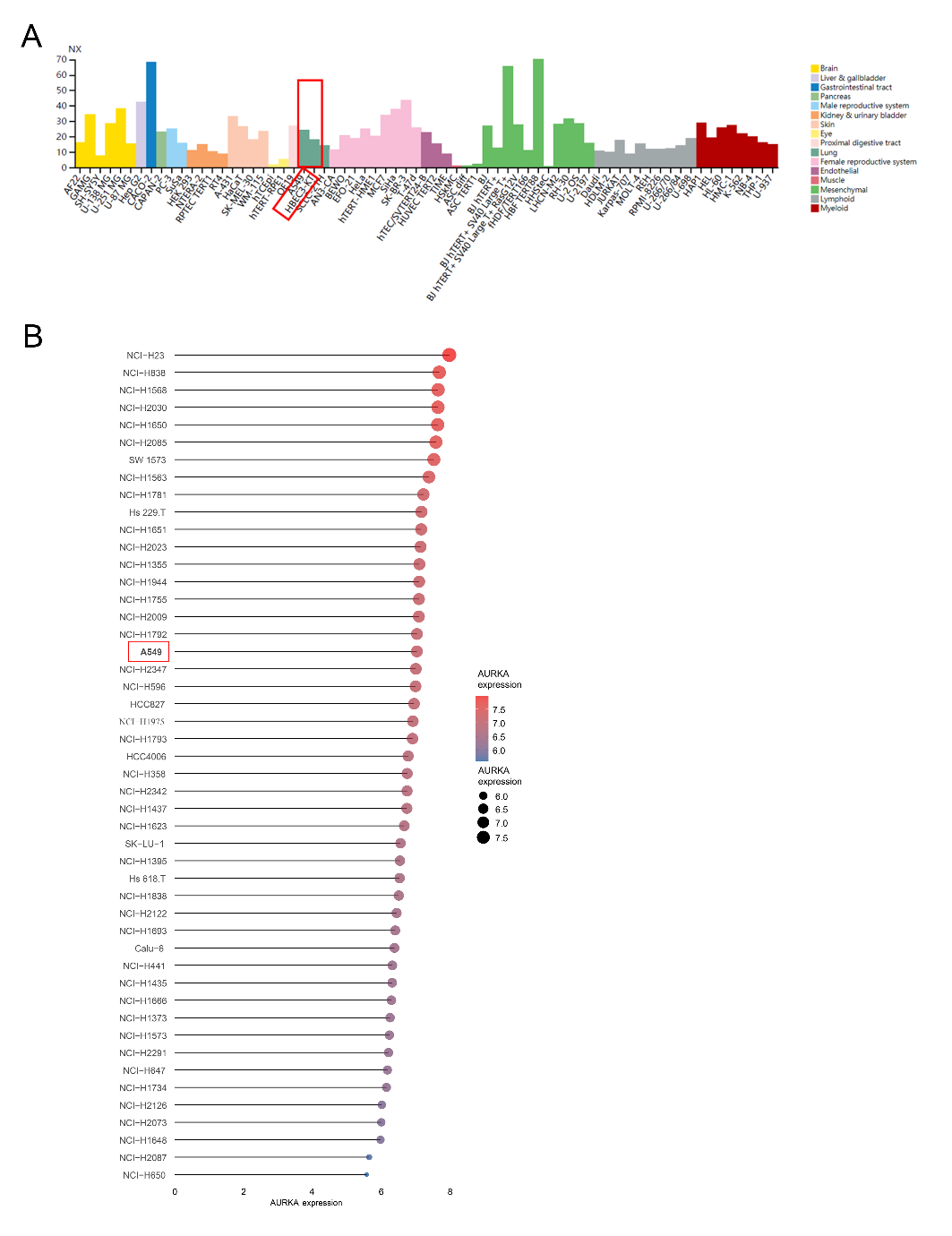
**

**Supplementary Figure 1. (A)** AURKA expression in tumor cell lines. **(B)** AURKA expression in LUAD cell lines.


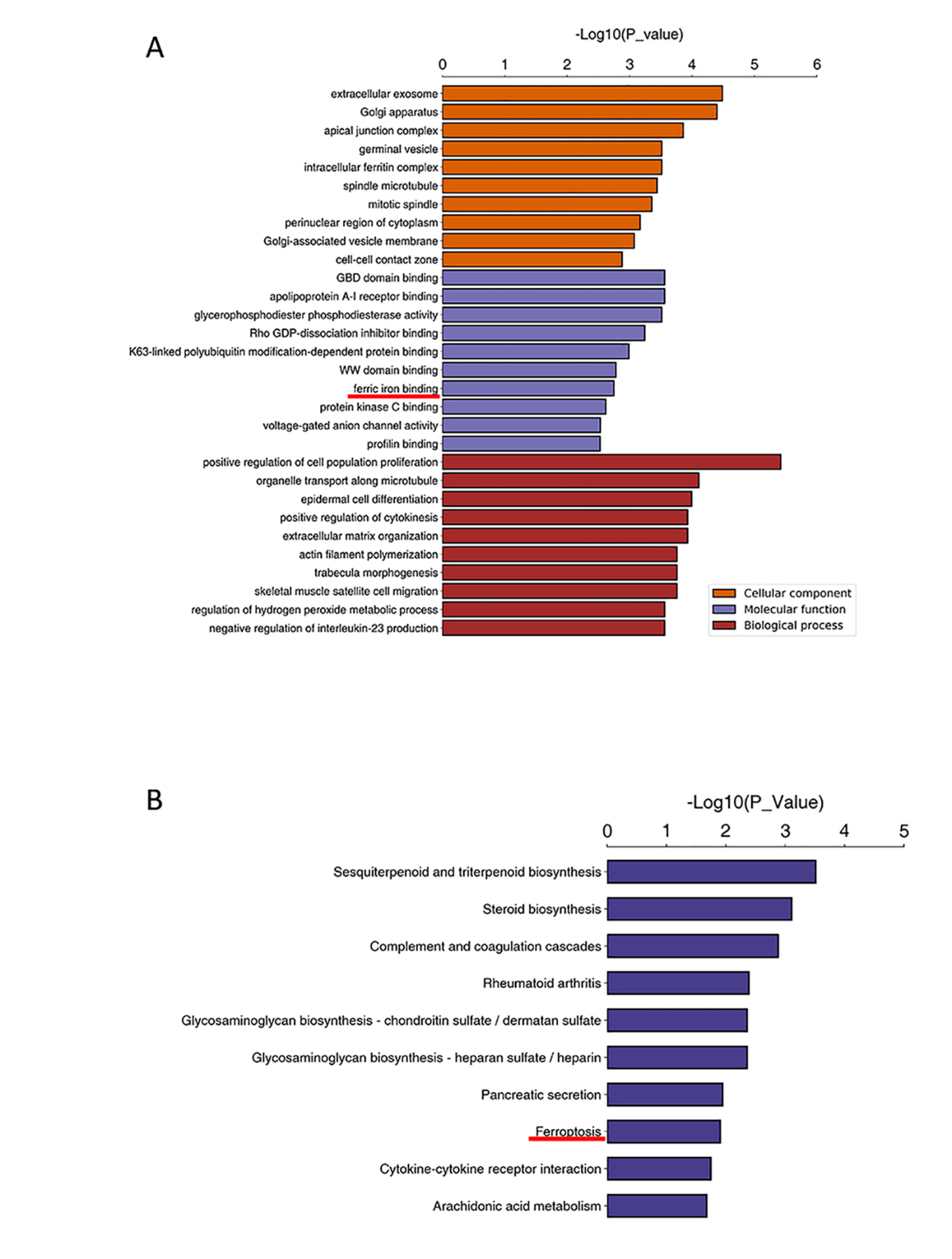


**Supplementary Figure 2.** **(A)** Protein GO terms analysis in A549 cells treated or not treated with OP-B for 24 h. **(B)** KEGG pathway analysis in A549 cells treated or not treated with OP-B for 24 h.
